# Supplementary material for: Hybrid Models and Biological Model Reduction with PyDSTool
Source: PLoS Comput Biol. 2012 Aug 9;8(8):e1002628. doi: 10.1371/journal.pcbi.1002628 (PMC3415397; doi:10.1371/journal.pcbi.1002628)
Supplement: Text S4 — Complete source code for the PyDSTool package (version 0.88.120504). Includes API documentation and help files linking to web pages. This file is identical to the current public release on Sourceforge.net. (ZIP) [file pcbi.1002628.s004.zip › PyDSTool/html/PyDSTool.Toolbox.optimizers.step.restart_conjugate_gradient.RestartPeriodicallyConjugateGradientStep-class.html]

xml version="1.0" encoding="ascii"?


PyDSTool.Toolbox.optimizers.step.restart\_conjugate\_gradient.RestartPeriodicallyConjugateGradientStep


| Home | Trees | Indices | Help | PyDSTool |
| --- | --- | --- | --- | --- |

Package PyDSTool ::
Package Toolbox ::
Package optimizers ::
Package step ::
Module restart\_conjugate\_gradient ::
Class RestartPeriodicallyConjugateGradientStep

[hide private]

[frames] | no frames]

# Class RestartPeriodicallyConjugateGradientStep

source code

```
object --+
         |
        RestartPeriodicallyConjugateGradientStep
```

---

A step decorator that periodically deletes the direction key in the
dictionary so that the CG search can be restarted


|  |  |
| --- | --- |
| Instance Methods | [hide private] |

|  |  |
| --- | --- |
| \_\_init\_\_(self, step, iteration\_period=10)  Constructs the decorator | source code |

|  |  |
| --- | --- |
| \_\_call\_\_(self, function, point, state)  Computes a step based on a function and a point | source code |

**Inherited from `object`**:
`__delattr__`,
`__getattribute__`,
`__hash__`,
`__new__`,
`__reduce__`,
`__reduce_ex__`,
`__repr__`,
`__setattr__`,
`__str__`


|  |  |
| --- | --- |
| Properties | [hide private] |

**Inherited from `object`**:
`__class__`


|  |  |
| --- | --- |
| Method Details | [hide private] |

|  |  |
| --- | --- |
| \_\_init\_\_(self, step, iteration\_period=10)  *(Constructor)* | source code |

Constructs the decorator

- step is the step that will be decorated
- iteration\_period is the number of iteration between the deletion

Overrides: object.\_\_init\_\_

  


| Home | Trees | Indices | Help | PyDSTool |
| --- | --- | --- | --- | --- |

|  |  |
| --- | --- |
| Generated by Epydoc 3.0.1 on Fri May 4 15:24:09 2012 | http://epydoc.sourceforge.net |
